# Attenuated SIV causes persisting neuroinflammation in the absence of a chronic viral load and neurotoxic antiretroviral therapy

**DOI:** 10.1097/QAD.0000000000001178

**Published:** 2016-09-28

**Authors:** Deborah Ferguson, Sean Clarke, Neil Berry, Neil Almond

**Affiliations:** Division of Virology, National Institute of Biological Standards and Control, South Mimms, Hertfordshire, UK.

**Keywords:** acute infection, cynomolgus macaques, HIV-associated neurocognitive impairment, neuropathology, simian immunodeficiency virus, suppressed viral load

## Abstract

Using simian models, where SIV chronic viral loads are naturally controlled in the absence of potentially neurotoxic therapies, we investigated the neuropathological events occurring during times of suppressed viraemia and when these events were initiated.

**Design:**

Cynomolgus macaques were infected with SIV strains that are naturally controlled to low levels of chronic viraemia. Study 1: animals were maintained up to 300 days after inoculation and analysed for viral-induced neuropathology following sustained suppression of chronic viral loads. Study 2: initiation and development of lesion was examined following 3, 10, 21, or 125 days SIVmacC8 infection.

**Methods:**

Formalin-fixed, paraffin-embedded brain sections were analysed following immunohistochemical staining for simian immunodeficiency virus (SIV) (KK41), blood–brain barrier leakage (ZO-1, fibrinogen), apoptosis (active caspase 3), neuroinflammation [GFAP, cyclooxygenase (COX)-1, COX-2], microglia and macrophage (Iba-1, CD68, and CD16), oligodendrocytes (CNPase1), MHC class II expression, and T cells (CD3 and CD8). Replicating SIV was detected through in-situ hybridization.

**Results:**

Study 1: neuroinflammation was present despite prolonged suppressed viraemia. Study 2: attenuated SIV entered the brain rapidly triggering acute phase neuroinflammatory responses. These did not return to naive levels and GFAP and COX-2 responses continued to develop during a chronic phase with a suppressed viral load.

**Conclusion:**

Neuroinflammatory responses similar to those in HIV neurocognitively impaired patients are present within macaque brains during prolonged periods of suppressed SIV viral load and in the absence of potentially neurotoxic antiretroviral drugs. These responses, initiated during acute infection, do not resolve despite the lack of on-going peripheral viraemia to potentially reseed the brain.

## Introduction

Neuropathology and symptoms of central nervous disease are late complications of HIV-1 infection. Before widespread use of antiretroviral therapy (ART), the most severe form, HIV-1-associated dementia (HAD), developed in 20–30% of patients [1,2]. Although HAD has virtually disappeared, up to 40% of HIV-infected individuals whose peripheral viral loads are effectively managed with antiretroviral drugs still develop HIV-1-associated neurocognitive impairment (HIV-NCI) [3,4] that can greatly affect daily life, notably disrupting adherence to their drug regimen. Current drugs, while able to suppress peripheral viral load for many years, do not protect against development of neurocognitive impairment [5]. Unless the cause of this impairment is determined and treatments identified, it may present a real barrier to further extension of therapeutic protection of infected individuals.

Few studies have compared HIV-associated neuropathology pre and post-ART introduction [6–8]; however, a common feature is the presence of astrogliosis and microgliosis in the absence of detectable peripheral viral load. Triggers for this neuropathology and its cause remain unclear with possible causes including both direct/indirect effects of virus or antiretroviral drugs within the brain [5]. Effective control of viral replication within this organ is challenging because of difficulties transporting drugs across the blood–brain barrier (BBB) and potential neurotoxic effects of long-term ART exposure. Therefore, new treatment strategies targeting the specific lesion may be needed to manage these neurological complications.

As relevant clinical material is difficult to obtain, we have investigated the value of simian models to investigate the pathogenesis of HIV-NCI. Previous reports of neuropathology in simian models relate to models of accelerated disease with high persisting peripheral viral loads. For modelling of patients on virus-suppressive ART, this would appear inappropriate. We have previously reported cynomolgus macaques (*Macaca fascicularis*) develop distinct neuropathology following extended infection with the *Nef*-disrupted, attenuated virus SIVmacC8 [9] with the lesion including both astrogliosis and microgliosis despite low chronic peripheral viral loads below 100 RNA copies/ml [10,11].

In this report, we characterize further the neuropathology arising from a 43-week infection with SIVmacC8. In particular, we use complementary techniques to assess BBB integrity. Furthermore, using brain samples collected from macaques used in the longitudinal analysis of virus kinetics and host responses following SIVmacC8 infection [11], we investigate the kinetics of virus entry into neurological tissues at very early time points after infection and associated neuropathological consequences.

These data indicate attenuated SIVmacC8 enters neurological tissues within a few days of infection, coincidental with disruption of the BBB. Neuroinflammation that immediately responds to this invasion progresses 5–10 months following infection, despite the BBB now appearing intact. On-going neuropathological consequences of HIV, therefore, may not depend upon repeated central nervous system exposure to new waves of replicating virus entering from the blood. These data should be considered when developing new treatments to control, delay, or reverse HIV-NCI.

## Methods

### Ethics statement

Nonhuman primates were used in strict accordance with UK Home Office guidelines, under licence from the Home Office Secretary of State. National Institute for Biological Standards and Control (NIBSC) is governed by the Animals (Scientific Procedures) Act 1986, which complies with EC Directive 86/609 and performs under licence (PPL 80/1952) granted following review of licence procedures by the NIBSC Ethical Review Process. Macaques were purpose bred and group housed for the study duration, with daily feeding and water access *ad libitum*. Regular modifications to housing areas were made to further enrich the study environment. Animals were acclimatized to their environment and deemed healthy by the named veterinary surgeon prior to study inclusion.

Animals were sedated for bleeding or virus inoculation by venepuncture. Frequent checks were made and unexpected changes in behaviour reviewed, including seeking veterinary advice where necessary. Regular blood samples were obtained to assess haematological parameters of incipient disease and veterinary advice sought when persisting abnormalities detected. The study was terminated and animals killed humanely by administering a ketamine anaesthetic overdose before development of overt symptomatic disease. All efforts were made to minimize animal suffering, including absence of nonessential procedures.

### Virus

SIVmacJ5 and SIVmacC8 are molecular clones derived from the 11/88 pool of SIVmac32H [10]. SIVmacC8 exhibits an attenuated phenotype in rhesus and cynomolgus macaques [10,12–14]. SIVmac17E-Fr was derived from SIVmac239 [15] and provided by JE Clements (Johns Hopkins School of Medicine, Baltimore).

### Experimental design

Study 1: As previously reported [9], two groups of four individuals were inoculated intravenously with either 5 × 10^3^ median tissue culture infectious dose (TCD_50_) SIVmacC8 or 10 MID_50_ SIVmacJ5. Total 20 weeks following this initial inoculation, both groups were inoculated with 50 TCD_50_ SIVmac17E-Fr. A third group of SIV-naive animals were challenged with SIVmac17E-Fr as controls. All animals were killed 23 weeks after SIVmac17E-Fr challenge.

Study 2: As previously reported [11], 10 cynomolgus macaques were inoculated intravenously with 5 × 10^3^ TCD_50_ 9/90 pool SIVmacC8. Pairs of animals were killed on days 3, 10, 21, and 125 post challenge (dpc). Two naive animals were killed for age-matched control tissues.

### Virological assessments

Plasma SIV viral RNA loads were determined by quantitative real time PCR as previously described [16].

### Tissue preparation

Whole brains were harvested no longer than 1 h after termination, fixed in 10% (volume/volume) formal saline for 4 weeks at 4^°^C and subsequently dissected [9].

### In-situ hybridization

In-situ hybridization was performed using digoxigenin (dig, Roche, Lewes, UK) labelled single-stranded DNA probes [17] detected via an alkaline phosphatase: 5-bromo-4-chloro-3’-indolyphosphate p-toluidine/ nitro-blue tetrazolium chloride chromogenic reaction (Roche) using a Leica Bond-Max-automated staining machine, utilizing the Research Mode option (Leica Microsystems, Wetzlar, Germany). Quantification of in-situ hybridization-positive cells was performed [9].

### Immunohistochemistry

Immunohistochemical analyses were performed using the Leica Bond-Max-automated stainer and Bond Polymer Refine staining system (Leica Microsystems). Antigen unmasking to allow antibody binding was undertaken using the optimal technique for each antibody/antigen combination. Tissue staining was graded by two independent experienced assessors and a mean score generated for each animal group. Box plots were generated using mean stained cells/mm^2^ grey matter [glial fibrillary acidic protein (GFAP)/ionized calcium binding adaptor molecule 1 (iba-1)] or maximum stained fibre length (GFAP) following manual counting of 10 independent images (frontal lobe, ×10 magnification). Median value within box, box defines middle 50% interquartile range, whiskers define upper/lower 25% of value distribution.

## Results

### Virological and haematological outcome of virus challenge

Outcome of study 1: virological and haematological outcomes of study 1 have been reported previously [9]; all four macaques inoculated with SIVmacC8 and all four inoculated with SIVmacJ5 became infected. Subsequent inoculation of these eight macaques with SIVmac17E-Fr did not result in evidence of superinfection by PCR or serology [18]. Naive macaques inoculated contemporaneously with SIVmac17E-Fr became productively infected. Between day 56 and 125 after SIVmac17E-Fr challenge, plasma viral load remained below the 1.3log_10_ level of detection.

No persisting alterations in CD4^+^, CD3^+^, and CD8^+^ lymphocytes or platelets were detected in peripheral blood [19].

Outcome of study 2: virological and haematological outcomes of study 2 have been reported previously [11,20]; following inoculation of SIVmacC8 all 10 macaques became infected and no persisting alterations in CD4^+^, [11], CD3^+^, and CD8^+^ lymphocytes or platelets were detected in peripheral blood (data not shown).

### Analysis of the blood–brain barrier following extended SIV infection

**Fig. 1:**
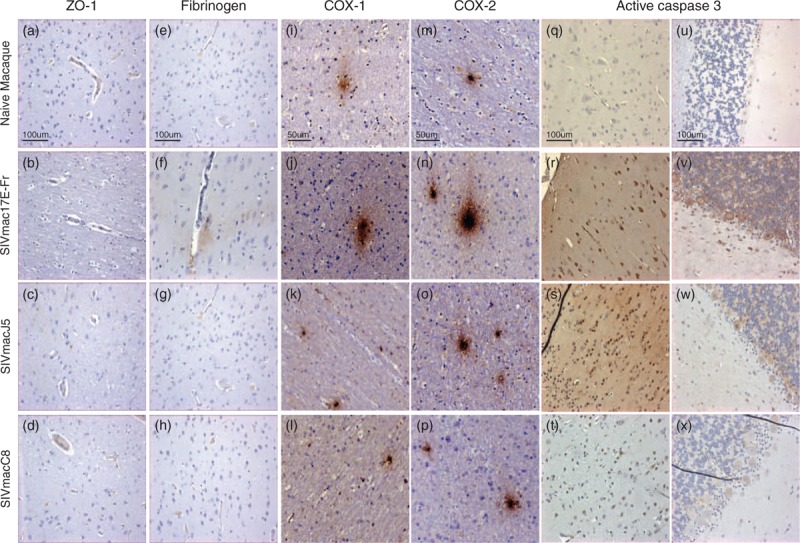
Representative images of the brain from SIV-naive or chronically infected cynomolgus macaques showing immunohistochemical staining results for (a–d) blood–brain barrier (ZO-1, ×20), (e–h) plasma leakage (fibrinogen ×20), inflammatory responses (i–l) COX-1 (×40), and (m–p) COX-2 (×40) and (q–x) apoptosis (active caspase 3 ×20) within (a–t) frontal lobe and (u–x) Purkinje fibres within the cerebellum.

We previously reported that brains of cynomolgus macaques infected with SIVmacC8 and SIVmacJ5 for 43 weeks, or SIVmac17E-Fr for 23 weeks harbour significant numbers of virus-infected cells [9]. To investigate whether this was a result of ready transit of virus and/or virus-infected cells into neurological tissue at that time, we assessed the integrity of the BBB using an antibody to Zonula occludens-1 (ZO-1) and the detection of fibrinogen leakage from blood vessels (Table 1; Fig. 1).

COX, cyclooxygenase.

Brain sections from SIV-naive animals exhibited consistent uninterrupted expression of ZO-1 within the endothelial walls of blood vessels in both grey and white matter areas. By contrast, at 23 weeks after infection with SIVmac17E-Fr, extensive loss of ZO-1 expression in both white and grey matter blood vessels was observed (Fig. 1b). Furthermore, immunostaining of adjacent tissue sections detected fibrinogen leakage from blood vessels into surrounding tissues of the cortical grey matter (Fig. 1f). At 43 weeks after infection with SIVmacJ5, there was a marked reduction in ZO-1 expression levels within both white and grey matter blood vessel walls (Fig. 1c). At 43 weeks after infection with SIVmacC8, patterns of ZO-1 staining were indistinguishable from those seen in SIV-naive controls with no evidence of fibrinogen leakage (Fig. 1a and d).

### Cyclooxygenase 1 expression in the brain

Immunohistochemical analysis of the inflammatory marker cyclooxygenase [(COX)-1; Table 1, Fig. 1] only detected baseline levels of expression in occasional cells within the both white and grey matter regions of cerebrum and brain stem sections from SIV-naive animals (Fig. 1i). All sections from SIVmac17E-Fr-infected macaques exhibited significant expression of COX-1 from both cells and blood vessels within mainly white matter (Fig. 1j) but also inclusive of some grey matter regions. Following either SIVmacJ5 or SIVmacC8, infection-elevated levels of cellular and blood vessel COX-1 expression were present within the white matter (Fig. 1k and l) and grey matter from all sections.

### Cyclooxygenase 2 expression in the brain

Immunohistochemical analysis of the inducible inflammatory marker COX-2 (Table 1, Fig. 1) detected baseline levels of expression within occasional cells in both white and grey matter regions of the cerebrum from SIV-naive animals (Fig. 1m). Brain sections from animals chronically infected with SIVmac17E-Fr exhibited strong expression of COX-2 in both white and grey matter of the cerebrum (Fig. 1n), midbrain, and brain stem with lower levels of expression within the cerebellum. Elevated levels of COX-2 expression were observed within these sections following either SIVmacJ5 (Fig. 1o) or SIVmacC8 infection (Fig. 1p).

### Caspase 3 expression in the brain

Immunohistochemical analysis for active caspase 3, a marker of apoptosis (Table 1, Fig. 1), within brain sections from SIV-naive controls detected low-level expression in glial cells of the cerebrum (Fig. 1q), midbrain, and brain stem. Minimal expression was detected within the cerebellum (Fig. 1u). Following 23 weeks, infection with SIVmac17E-Fr expression of active caspase 3 was greatly increased within both grey and white matter glial cells, particularly within the frontal lobe (Fig. 1r) and thalamus. Caspase 3 expression was also detected within neurones, notably Purkinje cells of the cerebellum (Fig. 1v). An identical pattern of caspase 3 expression was present in brain sections following 43 weeks infection with SIVmacJ5, although the intensity was slightly lower (Fig. 1l, s, and w). The distribution of active caspase 3 within brain sections from macaques chronically infected with SIVmacC8 was similar to that in SIV-naive controls (Fig. 1t and x).

### Kinetics of neuroinflammatory responses following infection with SIVmacC8

**Fig. 2:**
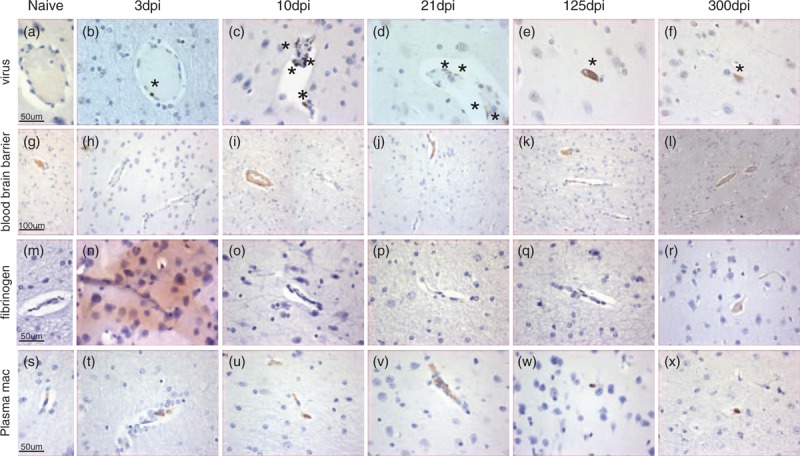
Representative images showing immunohistochemical staining results for (a–f) virus particles (stained particles highlighted∗ ×40), (g–l) blood–brain barrier (Z0–1, ×20), (m–r) plasma leakage (fibrinogen ×40), and (s–x) infiltrating macrophage (×40) within frontal lobe of either SIV-naive or SIV-infected brain samples during either acute or chronic infection.

As neuroinflammation following long-term infection with attenuated SIVmacC8 is evident, while the BBB appears intact, we investigated the early kinetics of neuroinvasion by SIVmacC8 (Table 2, Fig. 2). Immunohistochemical and in-situ hybridization analyses detected low numbers of SIV-infected cells by 3 days after infection that were closely associated with blood vessels (Fig. 2b). Increased numbers of SIV-positive cells were detected at 10 days after infection (Fig. 2c) with levels peaking 21 days after infection (Fig. 2d); these were reduced by 125 days after infection (Fig. 2e) when virus-positive cells were also located within grey matter.

Immunohistochemical staining for ZO-1 (Table 2, Fig. 2) 3 days after infection with SIVmacC8 revealed greatly reduced and intermittent expression within both white and grey matter blood vessels in the cerebrum (Fig. 2h). Within the thalamus, low levels of expression were restricted to large blood vessels. At 10 days after infection, ZO-1 expression in the cerebrum (Fig. 2i) and thalamus was high along endothelial cells of some larger white/grey matter blood vessels, whereas expression in smaller blood vessels remained low. At 21 days after infection, low-level ZO-1 expression was detectable along all cerebrum (Fig. 2j) and thalamus blood vessels. By 125 days after infection, ZO-1 expression levels were equivalent to SIV-naive brains (Fig. 2g and k).

Despite the intermittent ZO-1 staining, concurrent evidence for fibrinogen leakage from blood vessels was only detectable 3 days after SIVmacC8 infection and not subsequently (Fig. 2n–r).

### Detection of macrophages in the brain

From 3 days after infection with SIVmacC8 increased levels of CD68-expressing cells were present, predominantly associated with blood vessels within the cerebrum and midbrain. Levels peaked 21 days after infection and remained elevated thereafter (Table 2).

Similarly, the highest levels of CD16-expressing cells were present 3–21 days after infection around blood vessels in all regions of the brain analysed (Fig. 2t–v). At 125 and 300 days after infection occasional CD16-positive cells remained detectable, primarily in the cerebrum grey matter and not apparently associated with detectable blood vessels (Fig. 2w and x). SIV-naive controls did not harbour any detectable CD16-positive cells (Fig. 2s).

### Neuroinflammatory responses during acute infection with SIVmacC8

**Fig. 3:**
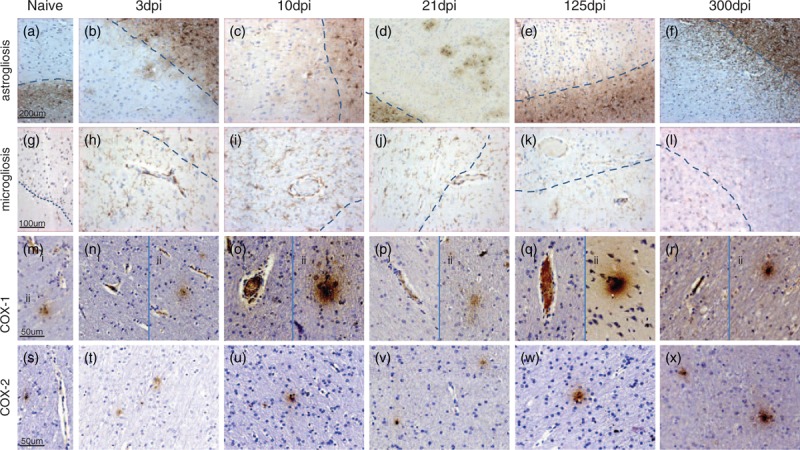
Representative images showing immunohistochemical staining results for (a–f) astrocytes (GFAP ×10), (g–l) microglia (Iba-1 ×20), and inflammatory responses (m–r) COX-1 (×40, panel i white matter blood vessels, panel ii white matter/grey matter cellular staining), and (s–x) COX-2 (×40) within frontal lobe of either SIV-naive or SIV-infected brain samples during either acute or chronic infection.

Sections of white matter from the brains from SIV-naive controls expressed low levels of both the astrocyte marker GFAP and microglial marker Iba-1 (Table 2, Fig. 3a and g). At 3 days after infection, GFAP expression in white matter increased, with staining located within astrocyte dendrites (Fig. 3b). Furthermore, astrocyte activation was detected around some grey matter blood vessels. Between 10 and 21 days after infection, increasingly frequent-activated astrocytes surrounding grey matter blood vessels were detected and the white/grey matter boundary became less distinct (Fig. 3c and d). At 125 days after infection, GFAP expression around individual blood vessels was no longer present; however, GFAP expression in the white matter remained elevated, with distinct GFAP-positive dendrites extending across into adjacent grey matter (Fig. 3e, Figure, Supplemental Digital Content 1, staining level box plot). Grey and white matter Iba-1 expression increased following SIV infection with high numbers of strongly stained microglial cell bodies and dendrites associated with blood vessel walls (Fig. 3h). Total 10–21 days after infection, microglial cell bodies remained strongly stained, whereas dendrites became truncated (Fig. 3i and j). Total 125 days after infection, Iba-1 cell body expression had reduced with extended dendrite networks again detectable. Grey matter Iba-1 expression remained well above SIV-naive tissue with the remaining strongly Iba-1-stained cell bodies associated with blood vessels (Fig. 3k, Figure, Supplemental Digital Content 2, staining level box plot).

COX, cyclooxygenase. Images a–l: dotted line indicates boundary between white and grey matter.

Expression of the inflammatory marker COX rapidly increased following infection with the two isoforms exhibiting differing patterns of induction (Table 2). Acute phase COX-1 responses within both blood vessels and some microglia were present by day 3 and peaked 10 days after infection (Fig. 3n and o). By day 21, elevated COX-1 was only present in microglia situated away from blood vessels (Fig. 3p). However, 125 days dpc, COX-1 expression in blood vessel walls and microglia (both proximal and distal to blood vessels) was again significantly raised and now mainly within grey matter (Fig. 3q). Levels remained above baseline at 300 days after infection (Fig. 3r). A small increase in the frequency of COX-2-expressing cells was seen up to 125 days after infection (Fig. 3t–w). However, by 300 days after infection, both the frequency of COX-2-expressing cells and their levels of expression had significantly increased (Fig. 3x).

High levels of 2’,3’-cyclic-nucleotide 3’-phosphodiesterase (CNPaseA1), a marker of oligodendrocytes, were present within both white and grey matter areas of SIV-naive brain sections (Table 2). However, immediately following SIVmacC8 infection a marked reduction in staining was observed within both the cerebrum and cerebellum. CNPaseA1 expression was reduced within the midbrain and brain stem but to a lesser degree. By 125 days after infection, expression of CNPaseA1 was returning to preinfection levels.

SIV-naive brain sections contained low levels of CD3^+^ T cells and cells-expressing major histocompatibility complex (MHC) class II. Immunostaining patterns for these markers did not alter following SIVmacC8 infection until 300 days after infection when CD8^+^ T cells became detectable (Table 2).

## Discussion

There have been few reports describing neuropathology following effective ART. Anthony *et al.* and Bell reported that while the incidence of HIV encephalitis, central nervous system opportunistic infections, and HAD have dramatically declined, on-going neuroinflammation persists. The clinical impact of this neuropathology is clearly evident [4,21,22]. However, the factors determining its development and the roles played by a persisting viral presence and host response remain unclear [1,2]. The paucity of pertinent clinical material, particularly during asymptomatic phases, would normally lead to the analysis of materials from model systems. However, most published simian studies investigating the neurological consequences of SIV infection are designed to mimic neuro-AIDS [15–26]. This does not reflect the clinical situation that we now need to model.

Our previously published work [9] identified astrogliosis and microgliosis, similar to that seen clinically [4,7] within the brains of cynomolgus macaques chronically infected with attenuated SIVmacC8. This led us to further investigate whether this neuropathology is dependent upon contemporaneous breakdown of the BBB; a structure crucial in regulating traffic in and out of the brain [27].

ZO-1 staining has been used to demonstrate loss of tight junctions and increasing permeability of the BBB in postmortem samples from patients with HIV encephalitis [28] and rhesus macaques with terminal AIDS [29]. Loss of ZO-1 has been associated with an accumulation of perivascular macrophage [30] and leakage of plasma proteins such as fibrinogen [31,32]. Our study demonstrates that at 23 weeks after infection with neurotropic SIV strain SIVmac17E-Fr, there is marked breakdown of the BBB. By contrast, following 43 weeks infection with the attenuated virus SIVmacC8 there is not.

In the absence of contemporaneous BBB damage, we sought to investigate alternative mechanisms for the observed neuropathological changes. Three markers COX-1, COX-2, and active caspase 3 were used to discriminate different pathways of neuropathology arising from inflammation or apoptosis. COX-1 and 2 are crucial catalysts in the production of essential lipid mediators including prostaglandins [33,34] and elevated levels of COX and prostaglandins have been observed in a number of acute and chronic neurodegenerative diseases, such as Alzheimer's disease [35], Parkinson's disease [36], HIV [37], and SIV [38]. Furthermore, the presence of prostaglandins has been linked to breakdown of the BBB [39] and enhanced migration of monocyte-derived cells [40,41]. Following chronic infection, distinct patterns of elevated cyclooxygenase and apoptotic responses were observed for each SIV strain. Thus, combining the data presented in this study with our previously published data [9] reveals that relatively recent infection (43 weeks) with attenuated SIVmacC8 results in a persisting neuropathology hallmarked by astrogliosis and microgliosis despite the BBB appearing intact and viral loads being almost undetectable.

These observations led us to question when and how the virus entered this compartment and the kinetics of the observed neuropathology. To address these questions, we analysed brains collected following a study of the early lesion of SIVmacC8 [11]. SIV-infected cells were detected adjacent to blood vessels by 3 days after infection, a time when the peripheral viraemia is only just detectable [11]. Breakdown of the BBB, elevated expression of the pathological inflammatory marker COX-1, and elevated levels of CD68+ and CD16+ macrophages were also detected at this time. As the frequency of virus-infected cells in the brain increased through to 21 days after infection, an increasing neuropathology was also observed, mirroring peripheral virus replication kinetics at this time. However, the kinetics of virus replication and markers of neuropathology were not linked at later times.

By 125 days after infection, with peripheral and tissue viraemia at the limits of detection, some neuroinflammatory markers and lesion appeared resolved. However, higher levels of GFAP, Iba-1, and COX-1 expression, notably in grey matter, identified persisting neuroinflammation. Despite low peripheral viral loads, by 300 days after infection levels of astrogliosis, microgliosis, and COX-2 expression were increasing. Although animal numbers do not allow calculation of statistical significance, box plot analysis suggests a trend of increasing neuropathology, including two markers associated with HIV-NCI [6,7], despite chronic peripheral viral replication remaining under effective control.

These data indicate that, in this simian model, continued detectable peripheral replication of the virus may not be required to maintain neuropathological changes. If so, then how are these changes maintained. Renner *et al.*[42] reported activation of microglia in SIV-infected macaque brains occurred even when interacting macrophage were uninfected. This triggers a self-perpetuating repertoire of proinflammatory and neurotoxic chemo/cytokines recruiting further monocytes to the brain. The acute COX-1 response and subsequent elevation of COX-2 may cause further neurochemical damage arising from crucial prostaglandin production within neurones [43]. The need for persisting active viral replication to drive this neuroinflammation is further questioned as macaques with divergent set point loads but similar primary viraemia profiles [14,11] exhibit similar neuropathology.

The fundamental surprise of our study is that infection with a genetically attenuated SIV, in the absence of any confounding exposure to powerful and potentially neurotoxic antiretroviral drugs, initiates a progressive neuropathology. Although our observations raise many questions regarding the effects of early viral sequestration, this model is capable of addressing them. The development of a conditionally live attenuated SIV (SIVrtTA; [44]), that is dependent on the presence of doxycycline to replicate can, in the absence of antiretrovirals, address the role virus replication plays in progressive neuropathology. Alternatively, treatments that blunt the primary viraemia but not alter peripheral set point loads, such as partially protective HIV or SIV vaccines could establish the role of this initial virological insult on subsequent neuropathology. The outcomes of these experimental studies will provide a framework to improve the clinical management of HIV-NCI.

## Acknowledgements

The authors thank the technical and veterinary staff at NIBSC for the animal care.

This work was funded by grants G9025730 and G0600007 from the MRC.

D.F., N.B., and N.A. conceived and designed the experiments. D.F. and S.C. performed the experiments and analysed the data. D.F. and N.A. wrote the manuscript. All authors participated in editing and approving the final manuscript.

### Conflicts of interest

There are no conflicts of interest.

## Supplementary Material

Supplemental Digital Content

## Figures and Tables

**Table 1:** Alterations to blood–brain barrier integrity, plasma leakage, and inflammatory responses within macaque brains following chronic SIV infection.

	Blood brain barrier ZO-1	Fibrinogen	COX-1	COX-2	Apoptosis active caspase 3
Naive macaque					
Cerebrum	+++	LBV	±	±	±
Midbrain	+++	LBV	−ve	−ve	+
Brain stem	+++	LBV	±	−ve	+
Cerebellum	++	LBV	−ve	−ve	±
SIVmacC8					
Cerebrum	+++	LBV	+	++	+
Midbrain	+++	LBV	+	++	+
Brain stem	++	LBV	+	±	+
Cerebellum	+++	LBV	+	+	+
SIVmacJ5					
Cerebrum	+	LBV±	+	++	++
Midbrain	+	LBV	++	++	++
Brain stem	+	LBV	+	++	+
Cerebellum	+	LBV	+	+	++
SIVmac 17E-Fr					
Cerebrum	±	LBV+	++	+++	+++
Midbrain	±	LBV+	++	+++	+++
Brain stem	±	LBV+	+	+++	++
Cerebellum	±	LBV	++	++	+++

Following extended infection with either SIVmacC8; SIVmacJ5, or SIVmac17E-Fr sections of macaque brains were examined for evidence of loss of ZO-1 staining within blood vessel walls and any accompanying leakage of plasma into brain tissue. Inflammatory responses via changes in COX-1 and COX-2 expression levels and levels of apoptosis were also determined. Brain stem, pons and medulla oblongata sections; Cerebellum, cerebellum sections; Cerebrum, frontal, parietal, occipital, and temporal lobe sections; Midbrain, thalamus and midbrain sections. +++ Very strong staining in all sections examined. ++ Strong staining in all sections examined. + Staining in all sections examined. ± Staining in sections examined with some animals negative. −ve Negative in all sections examined. COX-1, cyclooxygenase-1; LBV, lumen of blood vessel.

**Table 2:** Pathological changes and immunological responses within macaque brains during acute and chronic phase SIVmacC8 infection.

	Virus ISH IHC	BBB ZO-1	Fibrinogen	COX-1	COX-2	Astrocytes (GFAP)	Microglia (Iba-1)	Macrophage (CD68) (CD16)	Oligodendrocyte (CNPase1)	MHC-II	T cells
Naive macaque											
Cerebrum	−ve −ve	+++	LBV	±	±	+	+	+ −ve	+++	+	+ (CD3)
Midbrain	−ve −ve	+++	LBV	−ve	−ve	+	+	+ −ve	+++	+	+ (CD3)
Cerebellum	−ve −ve	++	LBV	−ve	−ve	+	+	−ve −ve	+++	+	+ (CD3)
SIVmacC8											
3 dpi											
Cerebrum	+ bv + bv	−ve	LBV +	+	+	++ bv	++	+/++ + bv	+	+	+ (CD3)
Midbrain	+ bv + bv	−ve	LBV	+	+	+++	++	+/++ + bv	++	+	+ (CD3)
Cerebellum	+ bv + bv		LBV			+++	++	+ −ve	+	+	+ (CD3)
10 dpi											
Cerebrum	+ bv + bv	±	LBV	++	+	+++ bv	+++	++ +++ bv	+	+	+ (CD3)
Midbrain	+ bv + bv	±	LBV	++	+	+++ bv	++++ bv	+++ +++ bv	++	+	+ (CD3)
Cerebellum	+ bv + bv		LBV						+	+	+ (CD3)
21 dpi											
Cerebrum	+ bv ++ bv	+	LBV	+	+	+++ bv	++ wm	+++ ++ bv	+	+	+ (CD3)
Midbrain	+ bv ++ bv	+	LBV	+	+	+++ bv	++ wm	+++ ++ bv	++	+	+ (CD3)
Cerebellum	+ bv ++ bv		LBV			+++	+++	+ −ve	+	+	+ (CD3)
125 dpi											
Cerebrum	+ +	++	LBV	++	+	+++	++++ bv	++ + bv + n	++	+	+ (CD3)
Midbrain	+ +	++	LBV	++	+	+++	+++ bv	+ + bv + n	+++	+	+ (CD3)
Cerebellum	+ +		LBV					+ −ve	++	+	+ (CD3)
300 dpi											
Cerebrum	+ +	+++	LBV	+	++	+++	++	++ + bv +n	+++	+	+ (CD3, CD8)
Midbrain	+ +	+++	LBV	+	++	+++	++	+ + bv +n	+++	+	+ (CD3, CD8)
Cerebellum	+ +	+++	LBV	+	+	+++	++	+ −ve	+++	+	+ (CD3, CD8)

Brain sections were examined for the presence of both replicating virus and viral particles, alterations to BBB integrity, inflammatory pathways, astrocytes, microglia, oligodendrocytes, MHC-II expression, and levels of infiltrating macrophage and T cells. bv, stained cells associated with blood vessels; Cerebellum, cerebellum sections; Cerebrum, frontal, parietal, occipital, and temporal lobe sections; LBV, lumen of blood vessel; midbrain, thalamus and midbrain sections; *n*, stained cells present within neuropil. BBB, blood–brain barrier; COX-1, cyclooxygenase-1; LBV, lumen of blood vessel; wm, white matter; +++, very strong staining in all sections examined; ++, strong staining in all sections examined; +, staining in all sections examined; ±, staining in sections examined with some animals negative; –ve, negative in all sections examined.
