# Supplementary material for: Attenuated SIV causes persisting neuroinflammation in the absence of a chronic viral load and neurotoxic antiretroviral therapy
Source: AIDS. 2016 Sep 28;30(16):2439–48. doi: 10.1097/QAD.0000000000001178 (PMC5051525; doi:10.1097/QAD.0000000000001178)
Supplement: Supplemental Digital Content [file aids-30-2439-s001.pdf]

Supplemental Figure S1a

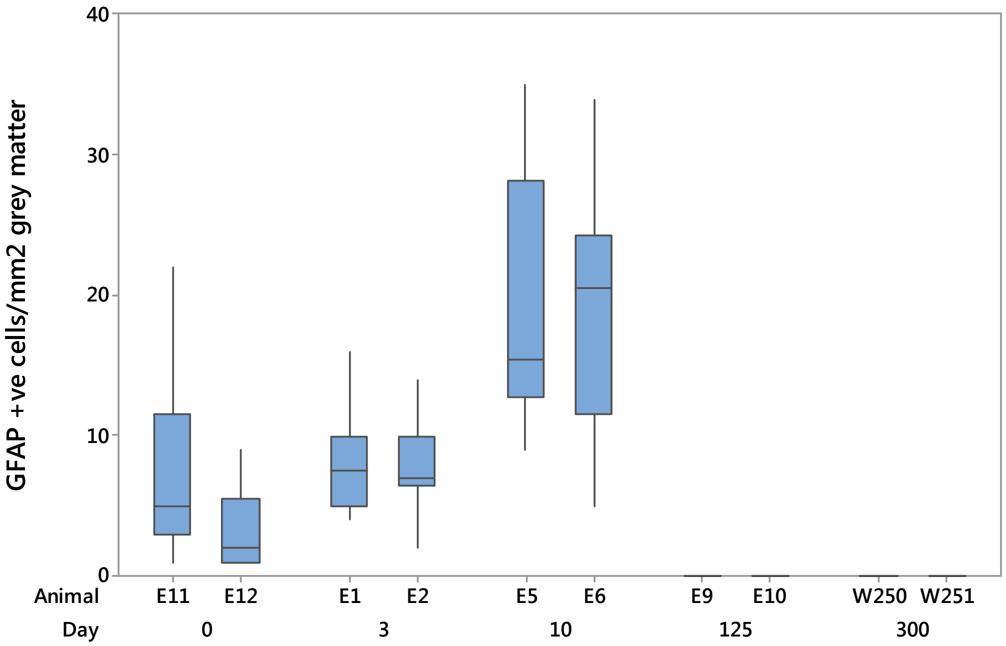

Supplemental Figure S1a : numbers of GFAP stained astrocyte cell bodies with associated stained dendrites within frontal lobe grey matter indicating a trend of increasing astrocyte activation within the grey matter during acute infection. Grey matter astrocyte cell body GFAP staining is absent at later time points.

Supplemental Figure S1b

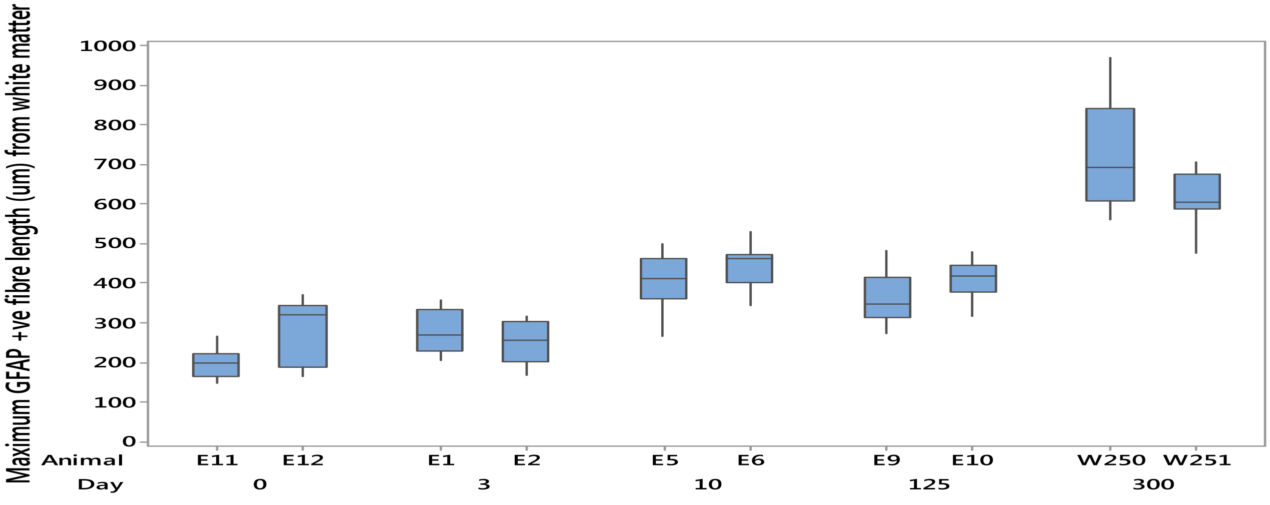

Supplemental Figure S1b: a pathology of increased extension of GFAP positive white matter astrocyte dendrites into the grey matter is present during chronic infection (300 days) despite control of peripheral viral load.

Mean number of stained cells per mm^2 or maximum stained fibre length from white matter/grey matter border were determined following manual counting of 10 independent images from each frontal lobe section at x10 magnification. Median value shown within box, box defines the middle 50% interquartile range, whiskers define upper and lower 25% of value distribution.

Supplemental Figure S2

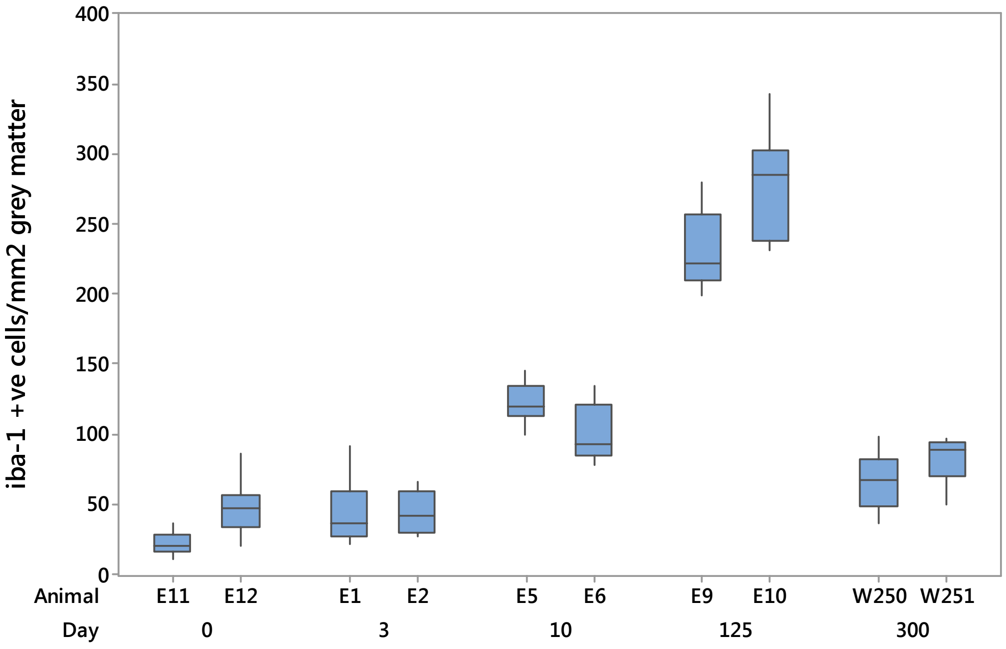

Supplemental Figure S2: numbers of iba-1 stained microglia cell bodies with associated stained dendrites within frontal lobe grey matter indicating a trend of increasing microglial activation within the grey matter over the first 125 days following SIVmacC8 infection and maintenance of elevated activation levels 300 days post infection despite control of peripheral viral load. Mean number of stained cells per mm^2 were determined following manual counting of 10 independent images from each frontal lobe section at x10 magnification. Median value shown within box, box defines the middle 50% interquartile range, whiskers define upper and lower 25% of value distribution.
